# A Real-World Analysis of Post-Marketing Surveillance Data Assessing the Incidence of Hyperkalemia or Acute Kidney Injury in Patients on Angiotensin-Converting Enzyme Inhibitors Versus Angiotensin-Receptor Blockers

**DOI:** 10.31083/j.rcm2404107

**Published:** 2023-04-13

**Authors:** Yining Wang, Qidong Ren, HuiTing Luo, Gang Chen, Bin Zhao, Xuemei Li

**Affiliations:** ^1^Nephrology Department, Peking Union Medical College Hospital, Peking Union Medical College, Chinese Academy of Medical Sciences, 100730 Beijing, China; ^2^Pharmacy Department, Peking Union Medical College Hospital, Peking Union Medical College, Chinese Academy of Medical Sciences, 100730 Beijing, China

**Keywords:** ACEI, ARB, hyperkalemia, acute kidney injury, FAERS database

## Abstract

**Background:**

The widely used Renin-angiotensin-aldosterone system inhibitor (RASI) may increase the risk of hyperkalemia and acute kidney injury (AKI). We aimed to analyze the RASI-related AKI or hyperkalemia reported in the Food and Drug Administration’s Adverse Event Reporting System (FAERS) database to optimize patients’ treatment and provide a reference for a clinically safe and rational prescription.

**Methods:**

We obtained data in FAERS recorded from January 2004 to December 2020. Disproportionality analysis and Bayesian analysis were used in data mining to screen the suspected AKI or hyperkalemia after RASI. The time to onset, hospitalization, and prognosis of RASI-associated AKI or hyperkalemia were also investigated.

**Results:**

We identified 11,301 RASI-related adverse events (AEs) of hyperkalemia and AKI in the FAERS database; 4997 were due to Angiotensin-converting enzyme inhibitors (ACEIs), 5658 were due to angiotensin receptor blockers (ARBs), and 646 were due to the combination of ACEI and ARB. AKI was more commonly reported in patients with ARB (78.42%) than ACEI users (57.27%). Hyperkalemia cases were reported more in ACEI users (28.70%) than ARB users (14.14%). The median time to onset of RAS-associated AKI was 135.0 (17.0–620.0) days. RASI-associated hyperkalemia occurred relatively later in ACEI users, with a median onset time of 261.0 (43.0–1097.7) days, compared with that of 200.5 (52.0–636.0) days in ARB users (*p *< 0.001). Among all AEs, 72.39% of cases received hospitalization. Death occurred in 6.3% of the renal AE cases. The elderly and heart failure were potential risk factors for death in patients who developed RASI-associated renal AEs, with an increased Odds Ratio (OR) compared with younger age (OR = 1.32) and hypertension patients (OR = 2.55). Based on the criteria of the four algorithms, the ACEI and ARB combination further increased the incidence of AKI and hyperkalemia, demonstrating the highest Reporting Odds Ratios (RORs), Proportional Reporting Ratios (PRRs) and Empirical Bayesian Geometric Average (EBGMs).

**Conclusions:**

Patients who indicated RASI for heart failure demonstrated a higher death risk when AEs occurred. ACEI combined with ARB can increase the incidence of hyperkalemia and AKI. Careful and individualized management is necessary.

## 1. Introduction 

Angiotensin-converting enzyme inhibitors (ACEIs) and angiotensin receptor blockers (ARBs) debuted in the early 1970s; they were first used as antihypertensive agents [1]. The indications have been gradually expanded to hypertensive heart failure, acute myocardial infarction, diabetic nephropathy, and non-diabetic nephropathy [2]. These interventions have demonstrated protective effects on cardiac and kidney function [3, 4]. ACEI or ARB monotherapies may fail to control proteinuria and blood pressure, which could be partly ascribed to the “aldosterone breakthrough” phenomenon [5]. To maximize the efficacy of the renin-angiotensin system (RAS) blockade, the dose of monotherapy was increased, or the combination of ACEI and ARB was applied; however such issues remained debatable [6, 7, 8]. ACEI, combined with ARB, is superior in reducing urine protein excretion [9, 10]. Although RAS inhibitors (RASI) benefits patients with hypertension, diabetes, heart failure, and chronic kidney diseases, they may increase the risk of hyperkalemia and acute kidney injury (AKI) [6, 11, 12, 13, 14].

Based on the Kidney Disease: Improving Global Outcomes 2021 (KDIGO 2021 guideline), RASI can be prescribed for hypertension, chronic kidney disease (CKD), and mild-to-moderate diabetes [15]. For patients prescribed with RASI, the incidence of AKI and hyperkalemia have been reported to vary in different diseases [8, 12, 16]. Moreover, the prescription of RASI could increase AKI incidence by 12% compared to patients without ACEI or ARB [17]. Hyperkalemia is more common in RASI-related adverse events (AEs), especially in patients taking the combination of ACEI and ARB, with an incidence rate of 10–20% [8]. Due to such AEs, the dual therapy of ACEI or ARB remained controversial. The Regulators in the United States (US), clinical guidelines, and some randomized controlled trials (RCTs) have suggested against the combination of ACEI and ARB therapies, especially in patients with diabetic nephropathy and CKD [7, 15, 18, 19, 20, 21]. However, clinical guidelines are inconsistent with real-world procedures; doctors still prescribe combination therapies when they encounter some cases such as refractory hypertension. We hope to apply real-world data to verify the risks of AKI and hyperkalemia when prescribing RASI combination therapies.

The Food and Drug Administration’s Adverse Event Reporting System (FAERS) is a database, which collects spontaneously reported drug AEs. The data volume herein is large, and the data type is diverse. Meanwhile, it is open to the public and often employed for signal mining AEs. Based on the FAERS system, we analyzed the RASI-related AKI and hyperkalemia in the real world.

## 2. Methods

### 2.1 Data Source

**Fig. 1.**
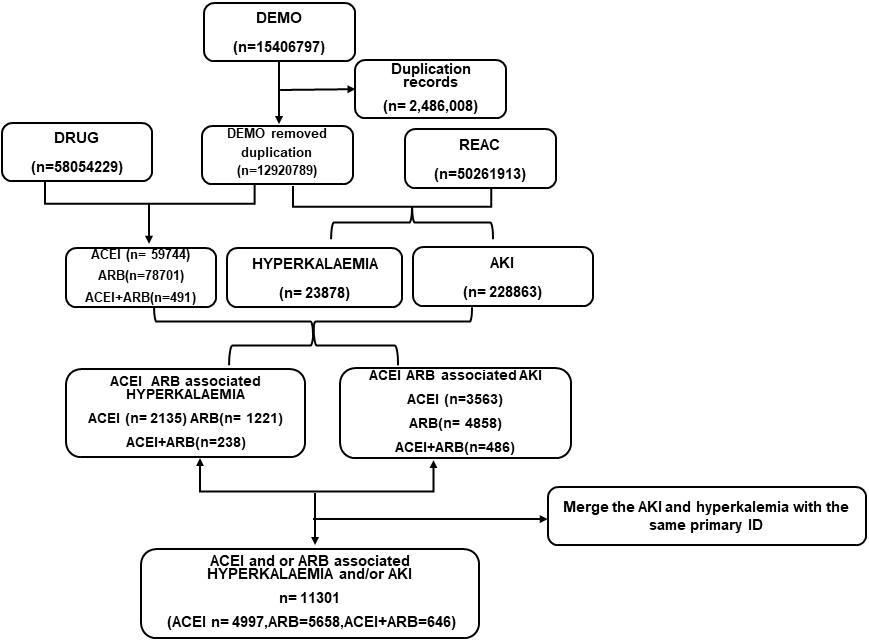
**Process of selection of cases of RASi-associated hyperkalemia and AKI from the FAERS database**. Abbreviation: ACEI, Angiotensin-converting enzyme inhibitor; ARB, angiotensin receptor blocker; DEMO, demographic and administrative information; AKI, acute kidney injury; DRUG, drug information; REAC, adverse events.

The FAERS database contains information on reports of adverse drug events and medication errors submitted by health professionals, patients, and manufacturers in the US and elsewhere. The FAERS data files comprise eight types of datasets as follows: patient demographic and administrative information (DEMO), drug information (DRUG), adverse events (REAC), patient outcomes (OUTC), report sources (RPSR), therapy start dates and end dates for reported drugs (THER), and indications for drug administration (INDI). In this study, we conducted a retrospective pharmacovigilance study using the FAERS database from January 2004 to December 2020 (Fig. 1). After we selected the RASI-associated hyperkalemia and RASI-associated AKI cases, we merged the records and generated RASI-associated hyperkalemia and/or AKI reports, using the PRIMARYID, which is the unique identifier for patients in the FAERS database.

### 2.2 Adverse Event and Drug Identification.

We selected “Hyperkalemia [100206462.3]” for further evaluation. Acute kidney injury was taken from the REAC files based on Medical Dictionary for Regulatory Activities (MedDRA, version 22.1, https://www.meddra.org/) at the Preferred Term level. We considered the following Preferred Terms as related to AKI, especially in the scenario when RASI was administered: “acute kidney injury [10069339]”, “subacute kidney injury [10081980]”, “blood creatinine increased [10005483]”, “blood urea abnormal [10005846]”, “glomerular filtration rate decreased [10018358]”, “renal impairment [10062237]”, “oliguria[10030302]”, “anuria [10002847]”, “dialysis [10061105]”, “renal tubular injury [10078933]”, “nephropathy toxic [10029155]”, “nephritis allergic [10029120]”, and “tubulointerstitial nephritis [10048302]”.

### 2.3 Data Mining

Based on Bayesian and nonproportional analysis, we carried out a disproportional analysis of FAERS using Reporting Odds Ratio (ROR), Proportional Reporting Ratio (PRR), and Empirical Bayesian Geometric Average (EBGM) to detect drug–ACEI or ARB pairs with higher-than-expected reporting rates compared with other drugs in the FDA registry [22, 23]. ROR, a signal is detected when the 95 percent double confidence limit exceeds 1 [24]. The PRR is a statistical tool for determining safety signals based on the percentage of specific ADRs of concern when all other medicines in the database are compared. The PRR and EBGM approaches use a proportional approach, taking advantage of the stable nature of an extensive database. The EBGM method is a quantitative approach to detect signals based on age, gender, and time and is less likely to generate false positive signals than PRR. The determination of the security signal’s presence and the signal’s intensity is based on three data: PRR (or EBGM), Chi-Square, or 95% confidence interval (CI) for PRR.

We also analyzed the time to onset of AKI or hyperkalemia related to RASIs, which was defined as the interval between the EVENT_DT (adverse event onset date) and the START_DT (start date of the RASIs administration).

Finally, we also evaluated the possible risk factors for death induced by RASI-associated AKI and hyperkalemia.

### 2.4 Statistical Analysis

We used descriptive analysis to summarize the clinical characteristics of AKI and hyperkalemia patients resulting in RASIs from the FAERS database. Nonparametric tests were used to compare the time to onset of different RASI-associated AKI and hyperkalemia (Mann-Whitney U test for dichotomous variables and Kruskal-Wallis’s test for more than two subgroups of respondents). Pearson’s chi-squared test or Fisher’s exact test compared the mortality and hospitalization rates between different prescriptions with ACEI with/or ARB. The statistical significance was set at *p *< 0.05 with 95% CI. All data mining and statistical analyses were conducted using SAS (version 9.4, SAS Institute Inc, Cary, NC, USA).

## 3. Results

### 3.1 Descriptive Analysis

We obtained 15,406,797 reports in the FAERS database from January 2004 to December 2020. After removing the 2,486,008 duplicated records and further data mining, we found 3594 hyperkalemia and 8907 AKI reports associated with RASI in the FAERS database (Fig. 1). We merged the hyperkalemia and AKI reports with the same primary ID and included 11,301 RASI-related AEs of hyperkalemia and AKI in Table 1. Among them, 4997 cases were due to ACEIs, 5658 patients were due to ARBs, and 646 patients were due to the combination of ACEI and ARB. Among the AEs, 68.20% of cases reported AKI, 21.18% reported hyperkalemia, and 10.62% of cases were complicated by both AKI and hyperkalemia. AKI (68.20%) was more commonly reported than hyperkalemia (21.18%) during RASI management, especially in patients prescribed with ARB (78.42%).

**Table 1.** **Clinical characteristics of patients with RASI-associated AKI and/or hyperkalemia sourced from the FAERS database (January 2004 to December 2020)**.

		Total	RAS inhibitors			*p*
			ACEI	ARB	Combination	
Number		11,301	4997	5658	646	
Adverse events, N (%)						<0.001*
	AKI	7707 (68.20%)	2862 (57.27%)	4437 (78.42%)	408 (63.16%)	
	Hyperkalemia	2394 (21.18%)	1434 (28.70%)	800 (14.14%)	160 (24.77%)	
	AKI and hyperkalemia	1200 (10.62%)	701 (14.03%)	421 (7.44%)	78 (12.07%)	
Gender, N (%)						<0.001*
	Male	5036 (56.81%)	2877 (62.58%)	1849 (49.70%)	310 (56.67%)	
	Female	3828 (43.19%)	1720 (37.42%)	1871 (50.30%)	237 (43.33%)	
Age, N, Mean ± SD		800, 970.28 ± 14.98	443, 070.47 ± 14.93	313, 371.00 ± 14.18	44, 663.36 ± 18.79	<0.001*
Age groups, N (%)						<0.001*
	<18	107 (1.34%)	53 (1.20%)	24 (0.77%)	30 (6.73%)	
	18–45	310 (3.87%)	168 (3.79%)	113 (3.61%)	29 (6.50%)	
	45–64	1918 (23.95%)	1099 (24.81%)	689 (21.99%)	130 (29.15%)	
	≥65	5674 (70.85%)	3110 (70.20%)	2307 (73.64%)	257 (57.62%)	
Reporting year, N (%)						<0.001*
	2004	534 (4.74%)	234 (4.72%)	259 (4.58%)	41 (6.36%)	
	2005	360 (3.20%)	167 (3.37%)	162 (2.87%)	31 (4.81%)	
	2006	352 (3.13%)	159 (3.21%)	167 (2.96%)	26 (4.03%)	
	2007	426 (3.79%)	220 (4.44%)	182 (3.22%)	24 (3.72%)	
	2008	426 (3.79%)	173 (3.49%)	230 (4.07%)	23 (3.57%)	
	2009	459 (4.08%)	188 (3.79%)	230 (4.07%)	41 (6.36%)	
	2010	431 (3.83%)	202 (4.08%)	205 (3.63%)	24 (3.72%)	
	2011	500 (4.44%)	241 (4.86%)	220 (3.90%)	39 (6.05%)	
	2012	501 (4.45%)	273 (5.51%)	205 (3.63%)	23 (3.57%)	
	2013	446 (3.96%)	255 (5.14%)	164 (2.90%)	27 (4.19%)	
	2014	541 (4.81%)	303 (6.11%)	170 (3.01%)	68 (10.54%)	
	2015	605 (5.38%)	340 (6.86%)	245 (4.34%)	20 (3.10%)	
	2016	886 (7.88%)	417 (8.41%)	409 (7.24%)	60 (9.30%)	
	2017	571 (5.08%)	292 (5.89%)	253 (4.48%)	26 (4.03%)	
	2018	1829 (16.26%)	600 (12.10%)	1150 (20.36%)	79 (12.25%)	
	2019	1898 (16.87%)	598 (12.06%)	1236 (21.88%)	64 (9.92%)	
	2020	485 (4.31%)	295 (5.95%)	161 (2.85%)	29 (4.50%)	
Reporter, N (%)						<0.001*
	Professional	8297 (81.91%)	4046 (90.11%)	3801 (74.72%)	450 (81.37%)	
	Non-professional	1833 (18.09%)	444 (9.89%)	1286 (25.28%)	103 (18.63%)	
Reporting areas, N (%)						<0.001*
	North America	3730 (34.86%)	1336 (28.09%)	2222 (41.48%)	172 (29.30%)	
	Europe	5633 (52.64%)	3001 (63.10%)	2327 (43.44%)	305 (51.96%)	
	Asia	974 (9.10%)	348 (7.32%)	538 (10.04%)	88 (14.99%)	
	South America	213 (1.99%)	17 (0.36%)	184 (3.43%)	12 (2.04%)	
	Oceania	137 (1.28%)	48 (1.01%)	79 (1.47%)	10 (1.70%)	
	Africa	13 (0.12%)	6 (0.13%)	7 (0.13%)	0 (0.00%)	
Indication, N (%)						<0.001*
	Hypertension	5984 (81.23%)	2384 (73.49%)	3287 (89.25%)	313 (71.14%)	
	Heart failure	536 (7.28%)	285 (8.79%)	216 (5.86%)	35 (7.95%)	
	Diabetes mellitus	66 (0.90%)	50 (1.54%)	16 (0.43%)	0 (0.00%)	
	Chronic kidney diseases	172 (2.33%)	89 (2.74%)	48 (1.30%)	35 (7.95%)	
	Coronary heart disease	138 (1.87%)	85 (2.62%)	31 (0.84%)	22 (5.00%)	
	Other cardiovascular diseases	127 (1.72%)	76 (2.34%)	29 (0.79%)	22 (5.00%)	
	Other indication	344 (4.67%)	275 (8.48%)	56 (1.52%)	13 (2.95%)	
AKI onset days, N, Median (Q1–Q3)		2, 677, 135.0 (17.0–620.0)	99, 357.0 (11.0–511.0)	1, 503, 196.0 (29.5–704.0)	181, 110.0 (24.0–481.0)	0.039
Hyperkalemia onset days, N, Median (Q1–Q3)		1, 469, 236.0 (46.0–908.0)	930, 261.0 (43.0–1097.75)	454, 200.5 (52.0–636.0)	85, 196.0 (83.0–924.0)	0.001*
Hospitalization, N (%)		8181 (72.39)	3717 (74.38)	4041 (71.42)	423 (65.48)	<0.001*
Death, N (%)		707 (6.3)	327 (6.5)	351 (6.2)	29 (4.5)	0.124
	AKI death, N (%)	588 (5.2)	260 (5.2)	302 (5.3)	26 (4.0)	0.363
	Hyperkalemia death, N (%)	181 (1.6)	107 (2.1)	66 (1.2)	8 (1.2)	<0.001*

*, *p *< 0.017 and was considered with significance among three-group comparisons. Abbreviations: RASI, renin-angiotensin system inhibitor; AKI, acute kidney injury; FAERS, Food and Drug Administration’s Adverse Event Reporting System; ACEI, angiotensin converting enzyme inhibitor; ARB, angiotensin receptor blocker.

Among all reported AEs, males contributed 56.71% of cases, and females contributed 43.19%. The elderly generation dominated the reported AEs, with 70.85% of subjects older than 65 years old, followed by 23.95% of cases aged 45–64 years old. We observed that the reporting volume of such AEs was relatively stable across the years, mainly between 3–5%, but recent years have indicated a clear growth trend. Especially in 2018 and 2019, the reporting AE percentage was >15% among all the cases. European reported the most cases with 5633 (52.64%), followed by North America (34.86%). While Asia, South America, Oceania, and Africa reported 9.10%, 1.99%, 1.28%, and 0.12%, respectively. RASIs were most indicated to hypertension, accounting for 81.23% of cases. Other indications included heart failure (7.28%), diabetes mellitus (0.90%), chronic kidney diseases (2.33%), coronary heart diseases (1.87%), other cardiovascular diseases (1.72%), and others (4.67%).

### 3.2 Prognosis of RASI-Associated Renal AEs

**Fig. 2.**
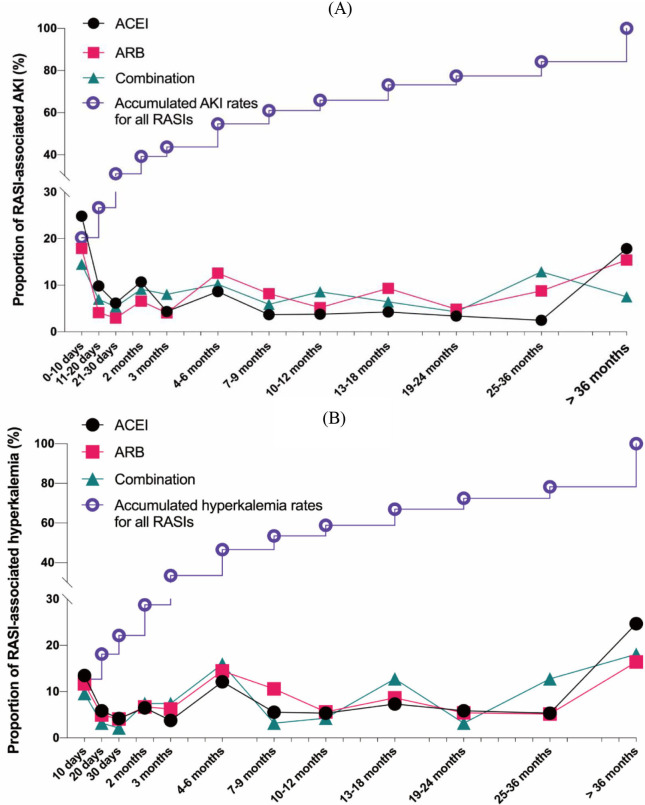
**Time to AKI or hyperkalemia onset**. (A) Time to AKI onset. (B) Time to hyperkalemia onset. AKI, acute kidney injury.

We described the onset time of AKI and hyperkalemia in Fig. 2. The median time to onset of RAS-associated AKI was 135.0 (17.0–620.0) days. ACEI induced AKI faster than ARB, with a median onset time of 57.0 (11.0–511.0) days compared with 196.0 (29.5–704.0) days (*p* = 0.039). In contrast, RASI-associated hyperkalemia occurred relatively later in ACEI users, with a median onset time of 261.0 (43.0–1097.7) days, compared with that of 200.5 (52.0–636.0) days in ARB users (*p *< 0.001). Among all reported AEs, 72.39% of cases received hospitalization, with the highest rate in patients who received ACEI monotherapies (74.38%). Death occurred in 6.3% of the renal AE population; among the 707 deceased cases, 327 received ACEI monotherapies, 351 received only ARB, and 29 received combination therapies (Table 1).

### 3.3 Evaluation of Possible Risk Factors for Death Induced by RAS-Associated AKI and Hyperkalemia

As shown in Table 2, old age and heart failure were potential risk factors for death in patients who developed RASI-associated renal AEs. The highest age group had a 1.32 times higher death risk than the lowest age group (*p* = 0.013). Patients indicated with RASI for heart failure developed a 2.55 times risk of death than the hypertension group (*p *< 0.001). The earlier onset of AKI and hyperkalemia could result in a higher possibility of death. Hospitalization reduced the death risk in AE patients to 62%, compared with those without hospitalization. We also detected the difference in death risk in different areas, with the lowest in North America and Oceania.

**Table 2.** **Evaluation of possible risk factors for death induced by RASI-associated AKI and/or hyperkalemia**.

		Statistics	Effect sizes (Odds ratio)	*p*-value (compared to the first categorical variables)
Gender				
	Male	5036 (56.81%)	1.0	
	Female	3828 (43.19%)	1.06 (0.90, 1.26)	0.474
Age (years), tertile				
	Low	2535 (31.65%)	1.0	
	Middle	2632 (32.86%)	1.06 (0.84, 1.33)	0.629
	High	2842 (35.49%)	1.32 (1.06, 1.64)	0.013*
Area				
	North America	3730 (34.86%)	1.0	
	Europe	5633 (52.64%)	1.95 (1.59, 2.38)	<0.001*
	Asia	974 (9.10%)	2.11 (1.57, 2.84)	<0.001*
	South America	213 (1.99%)	7.81 (5.42, 11.24)	<0.001*
	Oceania	137 (1.28%)	1.89 (0.94, 3.79)	0.074
	Africa	13 (0.12%)	8.05 (2.19, 29.59)	<0.01*
Indications				
	Hypertension	5984 (81.23%)	1.0	
	Heart failure	536 (7.28%)	2.55 (1.91, 3.41)	<0.001*
	Diabetes mellitus	66 (0.90%)	0.00 (0.00, inf.)	0.963
	Chronic kidney diseases	172 (2.33%)	0.58 (0.24, 1.43)	0.240
	Coronary heart disease	138 (1.87%)	1.20 (0.58, 2.47)	0.622
	Other cardiovascular diseases	127 (1.72%)	1.67 (0.86, 3.21)	0.127
Onset time to AKI, tertile				
	Short	887 (33.13%)	1.0	
	Medium	886 (33.10%)	0.97 (0.70, 1.36)	0.870
	Long	904 (33.77%)	0.62 (0.43, 0.90)	0.013*
Onset time to hyperkalemia, tertile				
	Short	490 (33.36%)	1.0	
	Medium	489 (33.29%)	0.81 (0.46, 1.43)	0.477
	Long	490 (33.36%)	0.52 (0.27, 0.99)	0.046*
Hospitalization				
	No	3120 (27.61%)	1.0	
	Yes	8181 (72.39%)	0.62 (0.53, 0.73)	<0.001*

*, *p *< 0.05 and was considered with significance. Abbreviations: RASI, renin-angiotensin system inhibitor; AKI, acute kidney injury.

### 3.4 Disproportionality Analysis and Bayesian Analysis

Based on the criteria of the four algorithms, we listed the renal AEs signals associated with RASI in Table 3. We detected the signals of AKI and hyperkalemia in the ACEI monotherapy, ARB monotherapy, and combination therapies. The AKI risk was similar in ACEI and ARB monotherapy; however, the risk increased with the combination therapies, based on their highest ROR, PRR, and EBGMs. It is worth noting that the risk of hyperkalemia associated with ACEI is much higher than that associated with ARB. The ACEI and ARB combination further increased the incidence of hyperkalemia based on its highest RORs, PRRs and EBGMs.

**Table 3.** **Detection of AKI and hyperkalemia signals for ACEI, ARB and the combination**.

Drug	AKI signals					Hyperkalemia signals				
	N	ROR	PRR	IC	EBGM	N	ROR	PRR	IC	EBGM
		(95% two-sided CI)	(χ^2^)	(IC025)	(EBGM05)		(95% two-sided CI)	(χ^2^)	(IC025)	(EBGM05)
ACEI	3563	3.56 (3.44, 3.68)	3.4 (6063.57)	1.75 (1.69)	3.37 (3.27)	2135	21.88 (20.92, 22.9)	21.14 (37366.95)	4.27 (4.08)	19.34 (18.62)
ARB	4858	3.71 (3.6, 3.82)	3.54 (8816.59)	1.8 (1.75)	3.48 (3.4)	1221	8.92 (8.41, 9.45)	8.79 (8017.43)	3.07 (2.9)	8.4 (8)
Combination	486	6.06 (5.52, 6.66)	5.56 (1847.74)	2.47 (2.25)	5.55 (5.13)	238	27.6 (24.21, 31.46)	26.32 (5749.36)	4.7 (4.13)	26.06 (23.36)

Abbreviations: AKI, acute kidney injury; ACEI, angiotensin converting enzyme inhibitor; ARB, angiotensin receptor blocker; N, the number of reports of RASi-associated AKI; ROR, reporting odds ratio; CI, confidence interval; PRR, proportional reporting ratio; χ^2^, chi-squared; IC, information component; EBGM, empirical Bayes geometric mean.

## 4. Discussion

Regarding the FAERS database, we summarized to date the largest real-world epidemiological characteristics and the risk factors for RASI-related AKI or hyperkalemia. We found that: (1) AKI is more commonly reported in ARB receivers than in patients with ACEI, whereas hyperkalemia is more often reported in ACEI receivers. (2) Patients indicated with RASI for heart failure demonstrated a higher death risk when AEs occurred. (3) ACEI combined with ARB may increase the risk of hyperkalemia and AKI, making it critical to manage a personalized approach in patient management.

The efficacy and safety issues of RASI were tested among large-scale RCTs [25, 26, 27]. Such RCTs could be very helpful; however, it was difficult to comprehensively evaluate the RASI AEs due to the relatively short study time, the lack of participants who were either too old or too young, the lack of endpoint event-judgment methods, and the multiple different factors of underlying diseases [28]. Our study is based on the FAERS’s extensive data analysis, which can effectively compensate for such defects as the small sample size and relatively short observation period in clinical trials.

RASI has intraglomerular effects, which can affect renal efferent arterioles and decrease the renal filtration pressure [29]. Our results indicated no noticeable difference in the risk signal intensity of AKI between ACEI and ARB, while the combination of both significantly increased the risk. A previous study showed that old age, deteriorated baseline renal functions, simultaneous loop diuretic treatment, and cardiac failure had been associated with AKI incidence during ACEI or ARB administration [30].

Previous studies have demonstrated that patients with a 30% increase in creatinine after RASI management were mainly middle-aged and elderly adults with cardio-renal comorbidity [31]. We also indicated that the factors of advanced age and heart diseases might associate with death induced by RASI-associated AKI or hyperkalemia. Therefore, it is suggested that RASI should be used cautiously and that creatinine levels should be closely monitored, especially in elderly patients with cardiovascular complications. Furthermore, our findings also indicated that hospitalization could reduce the death risk in AKI or hyperkalemia cases. Timely hospitalization could help such AE patients to recover better.

Hyperkalemia is another common complication of ACEI/ARB [32]. RASI causes hyperkalemia by affecting aldosterone production triggered by several major regulatory factors [29]. The renin-angiotensin-aldosterone system (RAAS) is a well-known regulator of blood pressure (BP); it also controls fluid and electrolyte balance through coordinated effects on the heart, blood vessels, and kidneys [33]. ACEI inhibits the transformation of angiotensin I (Ang I) into angiotensin II (Ang II). ARB only works on the AT1 receptor of Ang II, while Ang II is required to bind to the angiotensin II type 1 (AT1) receptor in blood vessels. When ARB is used, Ang II levels are elevated; while ACEI is used, Ang II is not secreted, which causes insufficient secretion of adrenal aldosterone [34]. We also found that hyperkalemia was more common in ACEI, which was consistent with such a mechanism. Researchers from the US reviewed electronic medical records, demonstrating that ACEI treatment was associated with a higher incidence and greater degree of hyperkalemia than ARB treatment, especially in CKD patients [33]. Interestingly, although hyperkalemia is common in hospitalized patients with acute heart failure, patients who maintained the dose of ACEI or ARBs during hospitalization had better 6-month survival after careful adjustment [16]. Another study also showed that the continuation of RASI might not associate with higher mortality in RASI-associated hyperkalemia after careful management [35]. Therefore, despite the risks, RASI can benefit certain patients if monitored appropriately.

Recently, double RAAS blockers are not recommended for patients with potential AEs, including renal dysfunction, hyperkalemia, and hypotension [18, 19, 36]. During clinical practice, clinicians may prescribe combination therapy when they encounter uncontrolled hypertension and heart failure. We also found that combination therapies increased the AKI signals by nearly two-fold, compared with ACEI or ARB monotherapies. However, there was no significant increase in mortality, which was consistent with the previous studies [37]. A meta-analysis revealed that dual therapy of ACEI and ARB further reduced urine protein excretion, and controlled blood pressure better [38]. Although the combination therapies may cause hypotension or hyperkalemia, individualized management, and proper potassium binders usage may extend such management in CKD patients [38]. We found that RASI increased the risk of death from AKI or hyperkalemia in patients with heart failure compared with hypertensive therapy (OR = 2.55). However, there was no increased death risk (OR = 0.58) in CKD patients compared to hypertensive patients who received RASI. Strategies to maintain RASI treatment after the onset of hyperkalemia may improve clinical outcomes in the CKD population [35]. We also observed the relationship between RASI-associated AEs and regional incidence. The results showed that North America had a higher incidence but a lower mortality rate, while Africa had the highest mortality rate. This bias could be ascribed to the difference in registered data from different countries. But it could not be very objective because such data was collected chiefly from America. The difference may be related to climate, customs, regional economic development, and medical conditions. In addition, we indicated that older patients, out-of-hospital treatment, and a shorter time to AEs onset after the prescription were related to increasing RASI-related mortality in this study.

Although this study managed the advantages of practical research and data mining technology, we admitted some limitations. First, in the data mining process, the database had incomplete information, such as the inputs being incorrect and the reports being preliminary, which could lead to a deviation from the analysis. Second, the available data in the database only involved patients with AEs. As there was no data on the total number of patients who received RASI and their baseline creatinine, we could not calculate the specific incidence of renal AEs after RASI or describe the severity of RASI-associated AKI.

## 5. Conclusions

We need to be alert for AKI and hyperkalemia when RASI is managed. AKI was more commonly reported in patients with ARB while hyperkalemia cases were more widely reported among ACEI users. Patients with heart failure had a significantly increased death risk from hyperkalemia or AKI after RASI use. The combination of ACEI and ARB increased the possibilities of hyperkalemia and AKI based on signals identified through the FAERS database. Therefore, careful and individualized management is necessary for such cases. We hope to further observe the risks and benefits of RASI combination therapies in real-world patients with CKD at different stages in the future.

## Data Availability

The data sets and resources analyzed during the current study are available from the corresponding author upon reasonable request.

## Abbreviations

RAAS, renin-angiotensin-aldosterone system; RASI, Renin-angiotensin-aldosterone system inhibitor; ACEI, Angiotensin-converting enzyme inhibitors; ARB, Angiotensin II receptor blockers; AKI, Acute Kidney Injury; AEs, Adverse events; RCTs, Randomized Controlled Trials.
